# Supplementary material for: Unit testing, model validation, and biological simulation
Source: F1000Res. 2016 Aug 10;5:1946. [Version 1] doi: 10.12688/f1000research.9315.1 (PMC5007758; doi:10.12688/f1000research.9315.1)
Supplement: Peer review history

# Peer review: Referee response for version 1

**DOI:** 10.5256/f1000research.10031.r15595

**Published:** 2016-08-31

**Authors:** Andrew Davison

**Affiliations:** 1 Unit of Neuroscience, Information et Complexity (UNIC), French National Center for Scientific Research, Gif-sur-Yvette, France

The article provides an introduction to automated software testing, its application to computational biology, and model validation as a form of testing, with examples taken from the OpenWorm project. The article is clearly written, and will be a helpful resource for computational biologists.

 The article could be improved by a deeper discussion of some of the more difficult issues in the automation of model validation:

- what criteria to apply when transforming a numerical measure of closeness into a pass/fail?
- how to support the use of different criteria by different scientists, who might weigh the relative importance of particular validations very differently?
- how to handle contradictory experimental results?

I would also be interested to read a discussion of possible improvements to continuous integration dashboards in the context of continuous validation, e.g. tracking the evolution of numerical validation results across model versions.

I have read this submission. I believe that I have an appropriate level of expertise to confirm that it is of an acceptable scientific standard.

# Peer review: Referee response for version 1

**DOI:** 10.5256/f1000research.10031.r15594

**Published:** 2016-08-30

**Authors:** Christian Roessert

**Affiliations:** 1 Blue Brain Project, École Polytechnique Fédérale de Lausanne (EPFL), Geneva, Switzerland

In this article the authors show how industrial practices of unit testing and test-driven development can be used and extended for computational modelling in biological sciences. The manuscript is well written and provides clear examples making it easy to understand the basic concepts.

 I believe that establishing a culture of test-driven development in biological sciences is of great importance. However, in my view applying software engineering practices to computational modelling is often not as easy as depicted by the authors. I have the following suggestions to improve the manuscript:

  

1. Judging the quality and validity of a computational model is a matter of scientific discussion and often cannot be easily reduced to a pass or fail decision in a model validation test. I would like to see a bit more detail on the transformation of the numeric score to a Boolean value used in the given ion channel test example but also on the general (statistical) concepts behind these decisions.
2. To iteratively improve a computational model, it is important to know not only if but also why a certain model fails or passes the model validation test. Since continuous integration systems are designed for simple verification tests: can the detailed results/figures and scores for each model validation test be shown directly on the CI dashboard? A discussion on the limits of current CI tools for biological modelling would be very helpful.
3. While the calculation of ion channel dynamics for a model validation test is computationally relatively cheap, computations become much more expensive once full detailed cell models or even networks have to be computed to validate against e.g. *in vivo* recordings. In these cases, the testing framework becomes much more sophisticated than “simple objects” and free services like Travis-CI will likely not be able to provide the required computational power. Is there a certain limit for your model validation test concept you would consider in the OpenWorm project and in general? Are there any ideas how to overcome these limitations? A discussion on the limits of the presented framework would be appreciated.

I have read this submission. I believe that I have an appropriate level of expertise to confirm that it is of an acceptable scientific standard, however I have significant reservations, as outlined above.

# Peer review: Referee response for version 1

**DOI:** 10.5256/f1000research.10031.r15597

**Published:** 2016-08-24

**Authors:** Robert Cannon

**Affiliations:** 1 Textensor Limited, Edinburgh, UK

The authors present an interesting review of how they have applied traditional software testing methodologies to the OpenWorm project. It provides a nicely balanced perspective on a subject that often leads to strong opinions.

 As they stay, the objective is to provide a focused case study of how testing is applied in the OpenWorm project. As such, the title is somewhat generic: I would suggest adding OpenWorm in there and possibly mentioning that this is a case study.

 Probably the most novel part of the work is the incorporation of "Model Validation Tests" which serve to verify that the components, such as ion channel models, from which the model is built, behave in line with experimental data. The authors state that "Ultimately, a scientific model is just another piece of software—thus it can be validated with respect to a specification." In a sense this is true, but, as Ref 10 points out ^1^ the specification in the literature is often vague, incomplete or generally erroneous.  The SciUnit "judge" method appears to be the answer to this, replacing the usual software testing "assert" function. Presumably a lot of the subtlety of the approach, and indeed the scientific input whether a model is indeed a good match to experiments, is embedded in the implementation of the various "judge" methods.  Although it is not essential for this paper it would be interesting to see a little more of how this is done in the OpenWorm project.

I have read this submission. I believe that I have an appropriate level of expertise to confirm that it is of an acceptable scientific standard.

## References

[1] De SchutterE: The Dangers of Plug-and-Play Simulation Using Shared Models. *Neuroinformatics*.2014; 10.1007/s12021-014-9224-7 10.1007/s12021-014-9224-7 24664409
